# Construction of a prediction model for rebleeding in patients with acute upper gastrointestinal bleeding

**DOI:** 10.1186/s40001-023-01349-3

**Published:** 2023-09-15

**Authors:** Yangping Zhuang, Shaohuai Xia, Junwei Chen, Jun Ke, Shirong Lin, Qingming Lin, Xiahong Tang, Hanlin Huang, Nan Zheng, Yi Wang, Feng Chen

**Affiliations:** 1 Shengli Clinical Medical College of Fujian Medical University, Fujian Medical University, Fuzhou, China; 2 Department of Emergency, The First Affiliated Hospital of Fujian Medical University, Fuzhou, Fujian China; 3 Department of Neurosurgery, Affiliated Hospital of Guilin Medical University, Guilin, 541001 Guangxi China; 4 Department of Emergency, The Affiliated Hospital of Putian University, Putian, Fujian China; 5 Department of Digestive Diseases, 900TH Hospital of Joint Logistics Support Force, Fuzhou, China; 6 Fujian Key Laboratory of Emergency Medicine, Fujian Provincial Hospital, Fuzhou, China

**Keywords:** Acute upper gastrointestinal bleeding, Rebleeding, AIMS65 score, Nomogram, Predictive model

## Abstract

**Background:**

The incidence of rebleeding in patients with upper gastrointestinal bleeding (UGIB) remains despite advances in intervention approaches. Therefore, early prediction of the risk of rebleeding could help to greatly reduce the mortality rate in these patients. We aim to develop and validate a new prediction model to predict the probability of rebleeding in patients with AUGIB.

**Methods:**

A total of 1170 AUGIB patients who completed the procedure of emergency gastroscopy within 48 h of admission were included. Logistic regression analyses were performed to construct a new prediction model. A receiver operating characteristic curve, a line graph, and a calibration and decision curve were used to assess the predictive performance of our new prediction model and compare its performance with that of the AIMS65 scoring system to determine the predictive value of our prediction model.

**Results:**

A new prediction model was constructed based on Lactic acid (LAC), neutrophil percentage (NEUTP), platelet (PLT), albumin (ALB), and D-DIMER. The AUC values and their 95% confidence interval (CI) for the new prediction model and the AIMS65 score were 0.746 and 0.619, respectively, and 0.697–0.795 and 0.567–0.670, respectively. In the training group, the C index values based on the prediction model and the AIMS65 scoring system were 0.720 and 0.610, respectively. In the validation group, the C index values based on the prediction model and the AIMS65 scoring system were 0.828 and 0.667, respectively. The decision and calibration curve analysis also showed that the prediction model was superior to the AIMS65 scoring system in terms of accuracy of prediction, consistency, and net clinical benefit.

**Conclusion:**

The prediction model can predict the probability of rebleeding in AUGIB patients after endoscopic hemostasis therapy.

## Introduction

Acute upper gastrointestinal bleeding (AUGIB) is one of the main reasons for visits to the emergency department [1, 2]. Approximately 50–150 individuals per 100,000 adult patients are admitted each year to the emergency department for symptoms related to upper gastrointestinal bleeding (UGIB) [2]. Peptic ulcer and esophagogastric varices are the most common causes of GIB, and approximately 45–60% of patients with AUGIB have peptic ulcer as the cause of their bleeding [3–5]. However, the highest mortality rate (3–14%) is observed for patients with AUGIB due to esophagogastric variceal hemorrhage and malignancy [6]. Despite advances in endoscopic techniques and the use of acid-suppressing drugs, the incidence of rebleeding in patients with UGIB remains at 7–16%. Rebleeding is a critical risk factor associated with high mortality, and it is one of the main prognostic indicators of AUGIB [7]. Therefore, early prediction of the risk of rebleeding could help to greatly reduce the mortality rate, and it can serve as a crucial approach for emergency management.

Presently, the Glasgow-Blatchford Score (GBS) [8] and the Rockall score [4] are the most widely used risk stratification tools for identifying patients with high risk UGIB. The GBS system has, however, not been widely used in routine clinical practice because it involves many variables with different weights. The Rockall score, which requires endoscopic data for score completion, cannot be used for patients without endoscopy. Therefore, both these scoring systems have limited clinical application in acute situations. The AIMS65 score is a new, unweighted risk stratification score with the advantages of simplicity and easy calculation, the AIM65 scoring was originally design to predict in hospital mortality [9]. It can be easily used in clinical practice and can be compared with laboratory values routinely obtained in emergency rooms. Some researchers have also proposed to use clinical indicators to predict the outcomes of patients with AUGIB, such as the presence of cancer, low total protein level, and high PDW, all of which were reported to predict 7-day rebleeding in patients with UGIB admitted to the emergency department [10].

There are several risk factors for UGIB, and many clinical risk scoring systems are available for risk stratification; however, differences in etiology, access to healthcare, and use of endoscopic approaches can lead to remarkable differences in patient outcomes. Hence, the risk scores need to be clinically validated internally. The present study aimed to investigate the relationship between a series of laboratory parameters and rebleeding in patients with AUGIB to determine the risk factors for rebleeding in these patients. These risk factors were used to construct a new predictive model, and the performance of the model was compared with that of the AIMS65 scoring system to provide a simple and effective tool for early detection of patients with rebleeding after endoscopic hemostasis treatment and to enable timely intervention.

## Materials and methods

### Patient selection

From October 2019 to July 2022, we retrospectively analyzed patients who visited the emergency department of Fujian Provincial Hospital, the First Affiliated Hospital of Fujian Medical University, and the Affiliated Hospital of Putian University, with hematemesis and melena as the primary symptoms and completed the procedure of emergency gastroscopy within 48 h of admission. A total of 1170 patients were diagnosed to have AUGIB. The inclusion criteria were as follows: (1) age > 14 years; (2) main clinical manifestations such as hematemesis, melena, or abdominal pain, with or without peripheral circulation disorders (dizziness, palpitation, amaurosis, transient syncope, shock, etc.), together with confirmation of the diagnosis of AUGIB on the basis of endoscopic findings and laboratory test results; and (3) patients who underwent endoscopic hemostasis within 48 h after entering the emergency room. The exclusion criteria were as follows: (1) bleeding caused by systemic diseases such as disseminated intravascular coagulation (DIC) and hematological diseases and (2) patients with incomplete case data and laboratory test results, and those who requested discharge from the hospital or refused to undergo gastroscopy.

Study groups: Following endoscopic hemostasis treatment, the patients were categorized into the rebleeding group and the non-rebleeding group based on the presence or absence of rebleeding.

The present study was approved by the Ethics Committee of Fujian Provincial Hospital and conducted in accordance with the Declaration of Helsinki. Because this was a retrospective study, the ethics committee waived off the requirement for patients to sign informed consent in accordance with national law and institutional guidelines. In this study, personal identifiable information of the enrolled patients was anonymized and replaced with a coding system.

### Data collection and definition

The following patient data were collected: (1) basic information: gender, age, medical history (including incidence of hypertension, diabetes, and coronary heart disease), and medication history (such as treatment with antiplatelet drugs and nonsteroidal anti-inflammatory drugs); (2) clinical symptoms and signs: with or without hematemesis and melena, systolic blood pressure, diastolic blood pressure, heart rate (HR), mean arterial pressure, and shock index at the time of visit; (3) laboratory indicators: the first test results after visit; all the tests were completed before endoscopy; (4) endoscopy results; endoscopic hemostasis and absence/presence of rebleeding during hospitalization after hemostatic treatment; (5) the AIMS65 score obtained before endoscopy.

Patients were considered to have rebleeding complication if they met any one of the following criteria: (1) hematemesis 24–48 h after endoscopy; (2) hemoglobin level ≥ 20 g/L or change in vital signs after hemostatic treatment and alteration in hemodynamic stability; (3) reappearance of black stools after the occurrence of yellow stools; (4) hematochezia after passing yellow stools or black stools; (5) sinus tachycardia (HR ≥ 110 beats/min) or hypotension (systolic blood pressure ≤ 90 mmHg) with no clear cause (e.g., sepsis, cardiogenic shock, or medication) after hemodynamic stability for ≥ 1 h; (6) after 2 consecutive stable hemoglobin values (hemoglobin difference < 5 g/L, interval > 3 h) Hemoglobin decreased ≥ 20 g/L; (7) continuous passage of black stool or appearance of blood in the stool, with hemoglobin level decreased by > 30 g/L within 24 h [11, 12].

All patients received an infusion of an intravenous proton pump inhibitor prior to endoscopy. The endoscopic procedure was performed within 48 h of the patient^’^s admission to the emergency department. The endoscopic treatment included epinephrine administration, high frequency coagulation, argon plasma coagulation, or application of vascular ligation.

### Statistical analysis

The study data were analyzed and processed using SPSS 21.0 software. The Kolmogorov–Smirnov method was used to assess the normality of the measurement data. Normally distributed data were expressed as mean ± standard deviation. Independent samples *t*-test was used for comparison between the groups. Non-normally distributed data were expressed as median and interquartile range. Nonparametric rank-sum test was used for comparison between the groups. Count data were expressed as numbers and percentages, and the chi-square test, adjusted chi-square test, or Fisher^**’**^s exact test were used for comparison of data between two groups. Univariate logistic regression was used to analyze the related risk factors for rebleeding. Variables with *P* < 0.05 in the univariate analysis were included in multivariate logistic regression, and the prediction model was constructed. The receiver operating characteristic (ROC) curve was drawn to evaluate the prediction value. R studio (version 4.2.0) was used to draw a nomogram, calculate the C index value, and draw the calibration graph and the decision curve analysis (DCA) graph. The XGBoost package in the R software was used for machine learning.

## Results

### General information

**Fig. 1:**
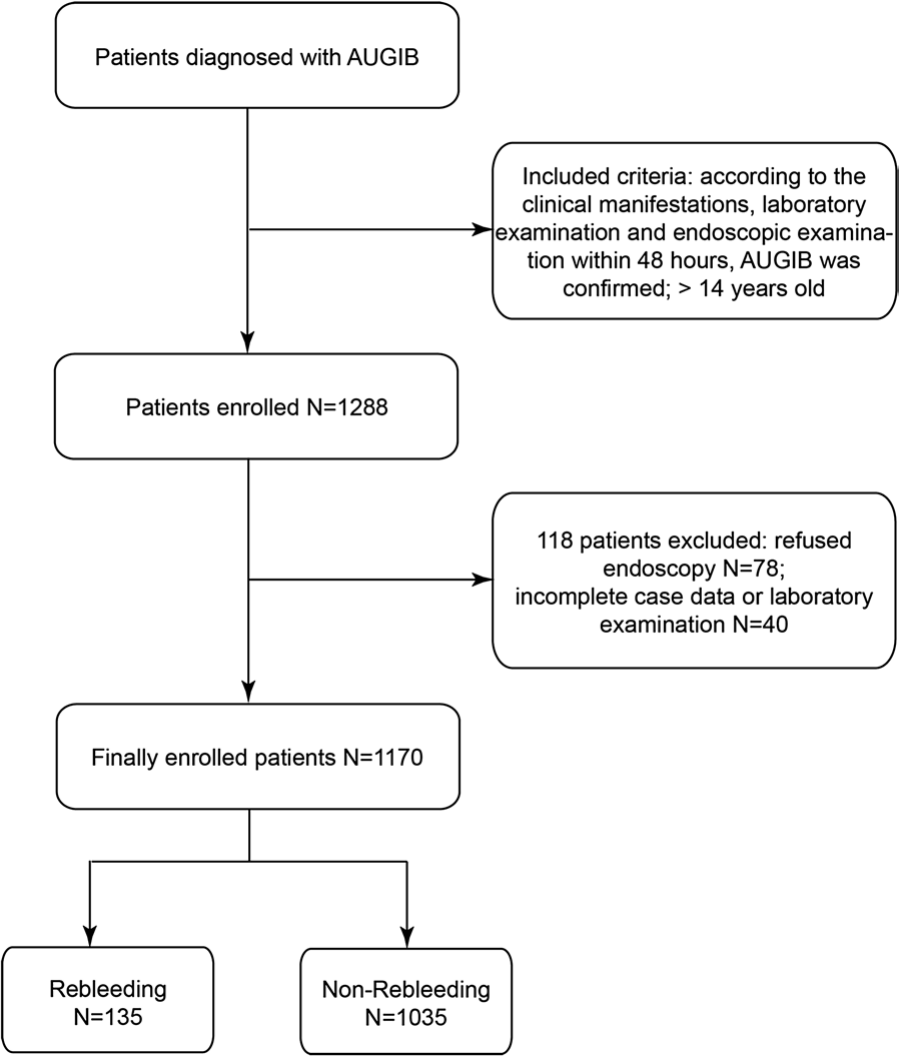
Flowchart for patients’ selection

The study enrolled 1288 patients with AUGIB who were admitted to the three large hospitals from October 2019 to July 2022. From these 1288 patients, 118 patients were excluded because they did not meet the inclusion criteria, and 1170 patients were finally included in the study, as shown in Fig. 1. The baseline data of enrolled patients are shown in Tables 1 and 2. Of the 1170 patients, 871 (74.4%) were males and 299 (25.6%) were females, with a male-to-female ratio of approximately 2.9:1. A total of 135 patients showed rebleeding after hemostatic treatment during hospitalization, and 1035 patients had no rebleeding. The proportion of patients with rebleeding was 11.5%. Eighty-seven (64.4%) patients in the rebleeding group vomited blood; this proportion was significantly higher (*P* < 0.05) than that in the non-rebleeding group (51.8%). Patients with a complaint of hematemesis were more likely to have rebleeding at the time of visit. The laboratory test results showed significant differences in lactic acid (LAC) level, neutrophil percentage (NEUTP), red blood cell distribution width (RDW), platelet (PLT) count, albumin (ALB) level, prothrombin time (PT), activated partial thromboplastin time (APTT), thrombin time (TT), fibrinogen (FIB) level, and D-DIMER level between the two groups (all *P* < 0.05). The rebleeding group had a higher AIMS65 score than the non-rebleeding group. While no differences were observed between the rebleeding group and non-rebleeding group concerning endoscopic performance and endoscopic hemostasis.

**Table 1:** Clinical characteristics of patients in the bleeding and non-rebleeding groups

Variables	No-rebleeding *n* = 1035 (88.5%)	Rebleeding *n* = 135 (11.5%)	*P*-value
Gender (male)	767 (74.1%)	104 (77%)	0.463
Age	56 (42, 67)	62 (53, 68)	0.004
Hematemesis	536 (51.8%)	87 (64.4%)	0.006
Melena	858 (82.9%)	107 (79.3%)	0.295
Medication history			
NSAIDs	115 (11.1%)	15 (11.1%)	1
Antiplatelet	45 (4.3%)	3 (2.2%)	0.242
Anticoagulants	19 (1.8%)	2 (1.4%)	0.771
Diabetes	132 (12.8%)	24 (17.8%)	0.106
Hypertension	276 (26.7%)	32 (23.7%)	0.462
Coronary heart disease	102 (9.9%)	12 (8.9%)	0.722
*Clinical data*			
Systolic blood pressure (mmHg)	115 (103, 128)	115 (103, 134)	0.924
Diastolic blood pressure (mmHg)	67 (60, 76)	66 (55, 80)	0.130
Heart rate (beats/min)	90 (78, 105)	92 (79, 105)	0.445
Mean arterial pressure(mmHg)	84 (75, 93)	81 (71, 99)	0.288
Shock index	0.78 (0.66, 0.93)	0.82 (0.64, 0.96)	0.398
*Lab results*			
LAC (mmol/L)	1.5 (1.0, 2.3)	1.9 (1.1, 3.4)	0.000
WBC (× 10^9^/L)	10 (7.5, 13)	9.3 (6.7, 12.7)	0.105
NEUTP (%)	73 (65, 82)	79.3 (71, 84)	0.000
RBC (× 10^12^/L)	2.9 (2.3, 3.7)	2.6 (2, 3.3)	0.001
HGB (g/L)	87 (67, 111)	81 (60, 98)	0.007
RDW (%)	13.5 (12.6, 16.2)	14.8 (13.6, 18.1)	0.000
PLT (× 10^9^/L)	222 (160, 273)	144 (100, 200)	0.000
PDW (%)	11.6 (10.1, 13.2)	12 (10.4, 15)	0.015
Alb (g/L)	34 (29, 38)	29 (24, 33)	0.000
BUN (mmol/L)	10.1 (6.3, 14.1)	9.7 (5.2, 12.6)	0.031
PT (sec)	12 (11.5, 13.2)	13.9 (12.2, 17)	0.000
APTT (sec)	23.5 (22, 25.8)	26.3 (22.8, 29.5)	0.000
TT (sec)	18.5 (17.5, 19.4)	19 (17.9, 20.4)	0.000
FIB (g/L)	2.04 (1.7, 2.57)	1.79 (1.3, 2.6)	0.000
D-DIMER (mg/L)	0.39 (0.19, 1.12)	0.98 (0.3, 3)	0.000
AIMS65 score	1 (0, 1)	1 (0, 2)	0.000

NSAIDs, non-steroidal anti-inflammatory drugs; LAC, lactic acid; WBC, white blood cell count; NEUTP, neutrophil percentage; RBC, red blood cell count; HGB, hemoglobin; RDW, red blood cell distribution width; PLT, platelet; PDW, platelet distribution width; Alb, albumin; BUN, blood urea nitrogen; PT, prothrombin time; APTT, activated partial thromboplastin time; TT, thrombin time; FIB, fibrinogen

**Table 2:** Endoscopy data and comparison between bleeding and non-rebleeding groups

Variables	No-rebleeding *n* = 1035 (88.5%)	Rebleeding *n* = 135 (11.5%)	*P*-value
Duodenal ulcer	475 (45.8%)	51 (3.7%)	0.075
Gastric ulcer	251 (24.2%)	33 (24.4%)	0.233
Esophageal varices	150 (14.4%)	27 (20%)	0.093
Gastric carcinoma	61 (5.8%)	12 (8.8)	0.176
Mallory–Weiss	41 (3.96%)	6 (4.4%)	0.788
Gastroduodenal anastomotic ulcer	22 (2.1%)	1 (0.7%)	0.276
Dieulafoy disease	12 (1.1%)	0	0.209
Esophageal carcinoma	9 (0.8%)	3 (2.2%)	0.142
Gastric stromal tumor	8 (0.7%)	0	0.305
Duodenal carcinoma	6 (0.5%)	2 (1.4%)	0.232
*Endoscopic hemostasis*			
Mechanical therapy	427 (41.2%)	52 (38.5%)	0.543
Combination therapy	231 (22.3%)	39 (28.8%)	0.088
Injection therapy	229 (22.1%)	20 (14.8%)	0.051
Electric coagulation	148 (14.2%)	24 (17.7%)	0.283

### Logistic regression analysis of risk factors for rebleeding in patients with AUGIB and construction of the prediction model

**Fig. 2:**
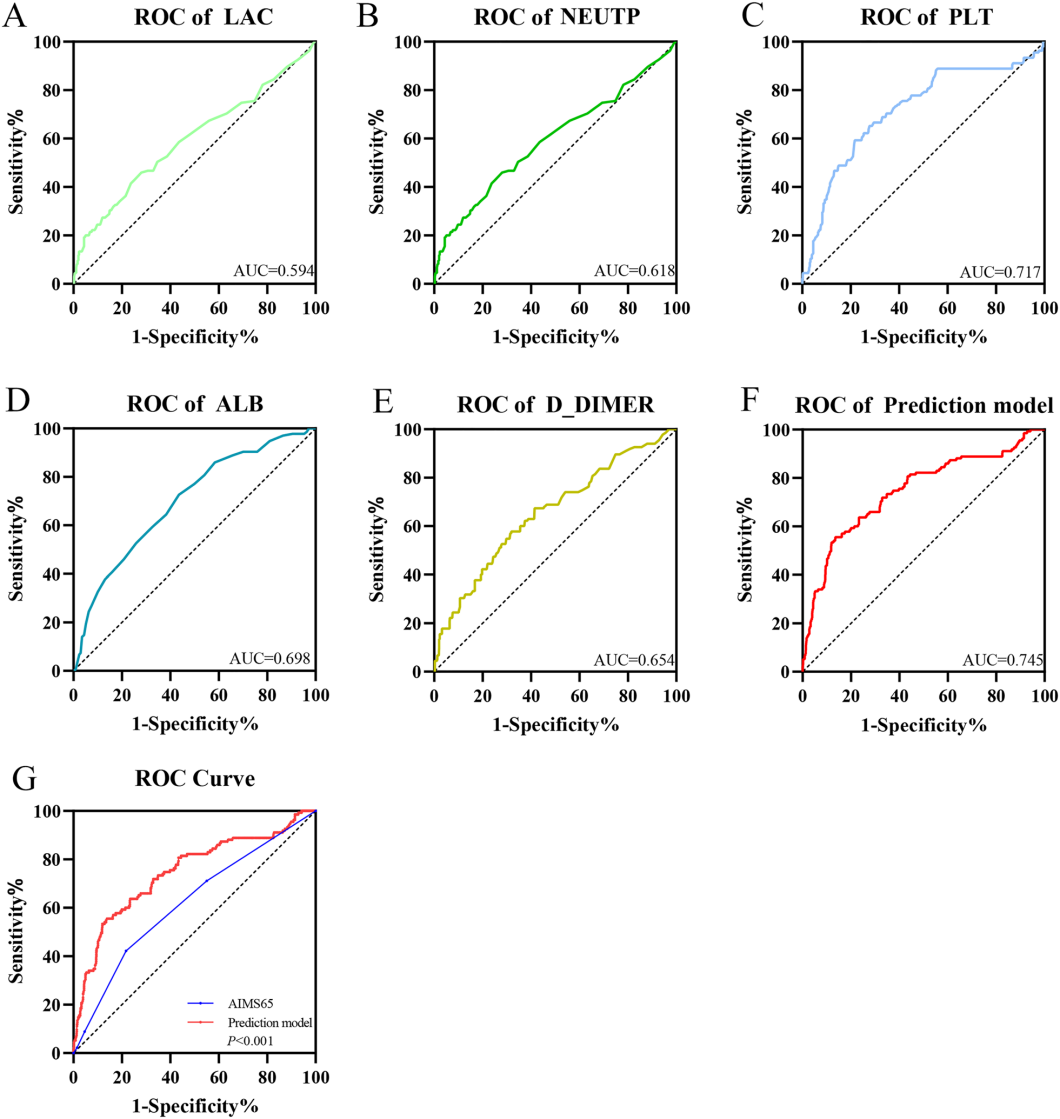
The ROC curves of LAC, NEUTP, PLT, ALB, D-DIMER, AIMS65 score, and the prediction model. **A** The area under the ROC curve (AUC) value of LAC was 0.594. **B** The AUC value of NEUTP was 0.618. **C** The AUC value of PLT was 0.717. **D** The AUC value of ALB was 0.698. **E** The AUC value of D-DIMER was 0.654. **F** The AUC value of the prediction model was 0.745. **G** Comparison of the ROC curves for the AIMS65 score and the prediction model

Univariate logistic regression analysis was performed with rebleeding of AUGIB patients as the dependent variable and the patients’ basic information, clinical symptoms and signs, and laboratory test indicators as the independent variables. According to the results, the following indicators were considered to be significant predictors (*P* < 0.05): age, hematemesis, LAC, NEUTP, RBC, HGB, RDW, PLT, PDW, ALB, PT, TT, FIB, and D-DIMER (Table 3). These indicators were then included in multivariate logistic regression analysis. As shown in Table 4, the indicators LAC, NEUTP, PLT, ALB, and D-DIMER were found to be significant predictors (*P* < 0.05), thus indicating that PLT and ALB were protective factors for evaluating rebleeding, while LAC, NEUTP, and D-DIMER were risk factors for rebleeding. These findings suggested that the lower the levels of PLT and ALB, the lower was the risk for rebleeding, while the higher the values of LAC, NEUTP, and D-DIMER, the higher was the possibility of rebleeding. A comparison of the area under the ROC curve (AUC) value of each index with the AIMS65 score (Fig. 2) showed that the AUC value of each index was lower than that of the AIMS65 score. Therefore, we combined these five indicators to construct a new prediction model. The regression equation was as follows: Logit P = − 1.253 + 0.099 LAC + 0.064 D-DIMER − 0.005 PLT + 0.023 NEUTP − 0.063 ALB. The optimal cut-off value was − 1.6, the sensitivity was 55.6%, and the specificity was 86.5%. The AUC values of the new prediction model and the classical AIMS65 score were compared. The AUC values and their 95% confidence interval (CI) for the new prediction model and the AIMS65 score were 0.746 and 0.619, respectively, and 0.697–0.795 and 0.567–0.670, respectively. A *t*-test showed a significant difference (*P* < 0.05) between the AUC values of the model and the AIMS65 score, thus indicating that the new prediction model had better prediction performance than the AIMS65 score (Fig. 2).

**Table 3:** Univariate logistic regression analysis of indicators of rebleeding

Variable	*B*	OR (95%CI)	*P*-value
Gender	0.159	1.172 (0.767–1.792)	0.463
Age	0.018	1.018 (1.006–1.030)	0.002
Hematemesis	0.523	1.687 (1.162–2.450)	0.006
Melena	− 0.238	0.788 (0.504–1.232)	0.296
Medication history	− 0.178	0.837 (0.507–1.383)	0.488
Diabetes	0.391	1.479 (0.918–2.384)	0.108
Hypertension	− 0.157	0.854 (0.561–1.300)	0.463
Coronary heart disease	− 0.114	0.892 (0.477–1.671)	0.722
Systolic blood pressure	− 0.001	0.999 (0.990–1.007)	0.760
Diastolic blood pressure	− 0.010	0.990 (0.977–1.004)	0.164
Mean arterial pressure	− 0.006	0.994 (0.982–1.006)	0.321
Heart rate	0.004	1.004 (0.994–1.014)	0.468
Shock index	0.307	1.359 (0.650–2.839)	0.415
LAC	0.155	1.168 (1.102–1.239)	0.000
WBC	− 0.031	0.969 (0.930–1.010)	0.138
NEUTP	0.029	1.030 (1.013–1.047)	0.001
RBC	− 0.356	0.700 (0.573–0.857)	0.001
HGB	− 0.010	0.990 (0.984–0.997)	0.003
RDW	0.101	1.106 (1.051–1.164)	0.000
PLT	− 0.008	0.992 (0.990–0.995)	0.000
PDW	0.090	1.094 (1.027–1.165)	0.005
ALB	− 0.109	0.897 (0.872–0.923)	0.000
BUN	− 0.008	0.992 (0.966–1.019)	0.541
PT	0.057	1.059 (1.032–1.086)	0.000
APTT	0.000	1.000 (0.998–1.002)	0.957
TT	0.109	1.115 (1.049–1.185)	0.000
FIB	− 0.332	0.718 (0.570–0.904)	0.005
D-DIMER	0.102	1.107 (1.058–1.159)	0.000
Endoscopic hemostasis	− 0.120	0.887 (0.738–1.066)	0.201

**Table 4:** Multivariate logistic regression analysis of indicators of rebleeding

Variable	*B*	OR (95%CI)	*P*-value
Age	0.000	1 (0.987–1.014)	0.981
Hematemesis	0.178	1.195 (0.773–1.848)	0.423
LAC	0.097	1.102 (1.035–1.173)	0.002
NEUTP	0.020	1.021 (1.002–1.039)	0.027
RBC	− 0.002	0.998 (0.633–1.575)	0.994
HGB	0.000	1 (0.984–1.016)	0.982
RDW	0.066	1.068 (0.995–1.146)	0.069
PLT	− 0.005	0.995 (0.992–0.998)	0.001
PDW	− 0.017	0.983 (0.916–1.054)	0.630
ALB	− 0.054	0.948 (0.910–0.987)	0.010
PT	0.021	1.021 (0.989–1.054)	0.197
TT	0.060	1.062 (0.985–1.146)	0.119
FIB	0.225	1.253 (0.946–1.660)	0.116
D-DIMER	0.063	1.065 (1.024–1.108)	0.002

### Comparison of the importance and model accuracy of each independent risk factor in the prediction model by machine learning

**Fig. 3:**
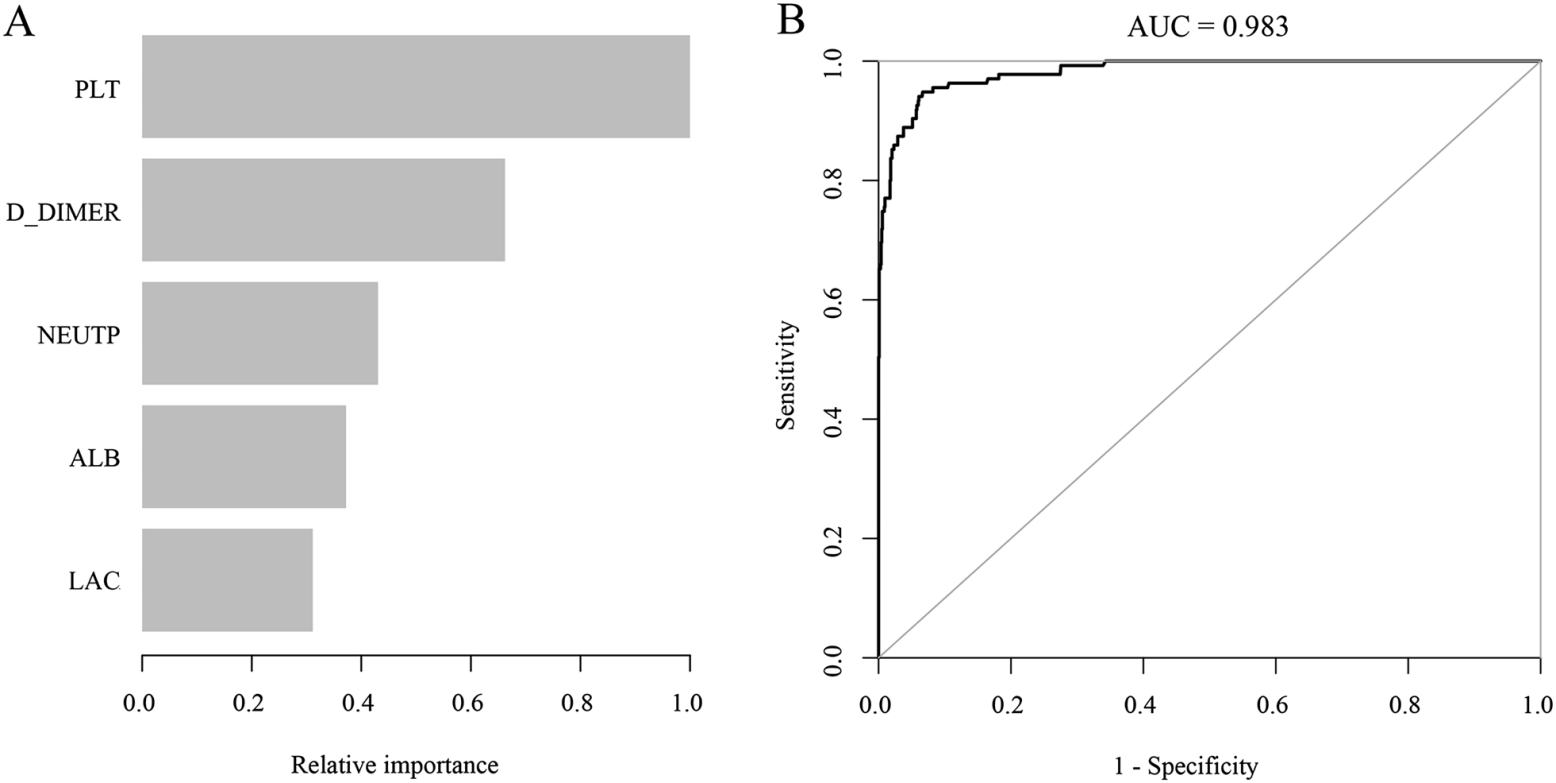
**A** The relative rankings of variables in the prediction model as determined by machine learning. **B** ROC curves of the prediction model as determined by machine learning

The independent risk factors (LAC, NEUTP, PLT, ALB, and D-DIMER) screened-out by the multivariate logistic risk regression model were ranked according to their importance by machine learning using R language. The ranking assigned by the new prediction model in the order of high to low was as follows: PLT > D-DIMER > NEUTP > ALB > LAC. Thus, as shown in Fig. 3A, B, the most important risk factor in the prediction model was PLT, followed by D-DIMER, and the AUC value of the prediction model was 0.983.

### Comparison of predictive performance between the training and validation groups using nomograms based on the predictive factors

**Fig. 4:**
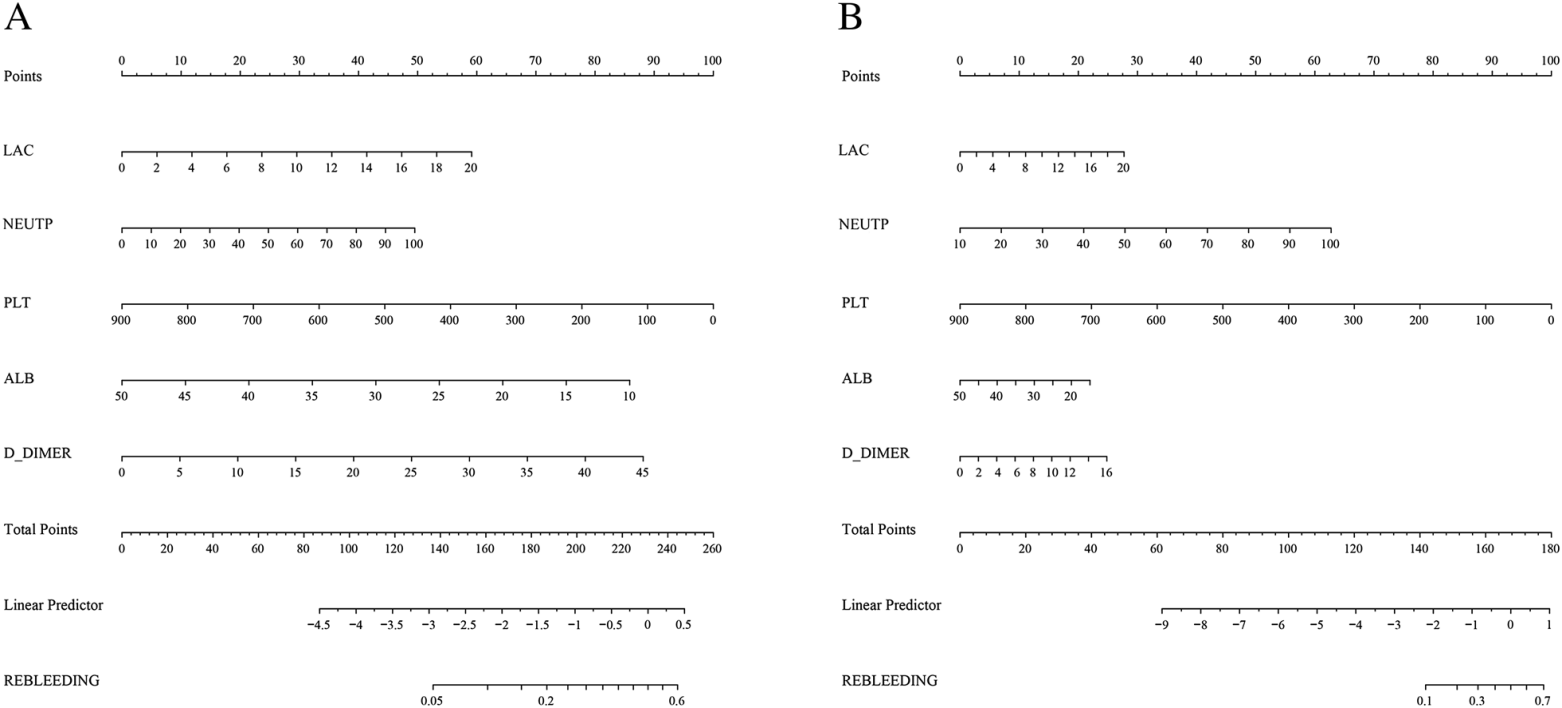
A nomogram was drawn using the risk factors LAC, NEUTP, PLT, ALB, and D-DIMER to predict rebleeding in AUGIB patients, followed by random splitting into the training group (**A**) and the validation group (**B**) in a 3:1 ratio

**Fig. 5:**
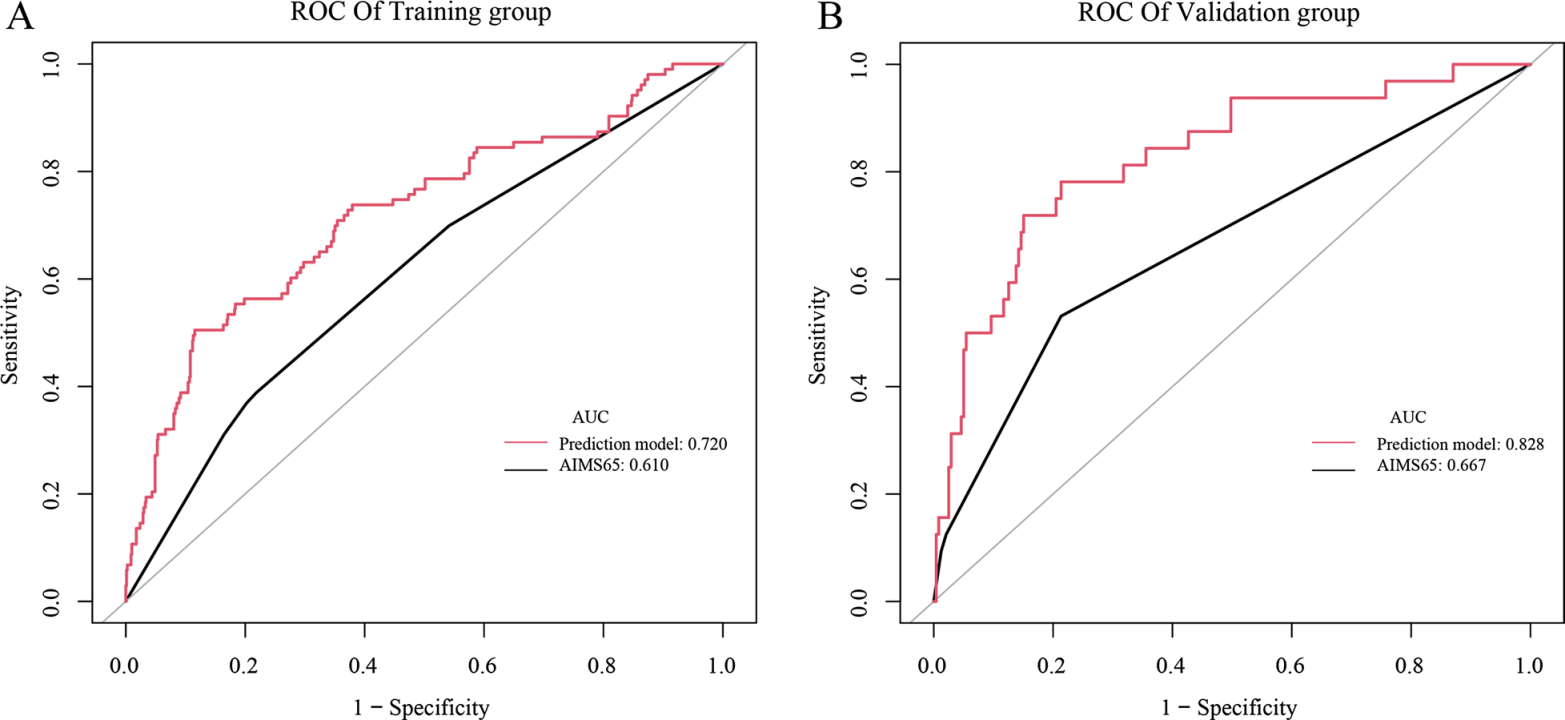
The C index values of the AIMS65 score system and the prediction model for the training and validation groups. (**A**) The C index values of the AIMS65 score system and the prediction model for the training group were 0.61 and 0.72, respectively. (**B**) The C index values of the AIMS65 score system and the prediction model for the validation group were 0.667 and 0.828, respectively

**Fig. 6:**
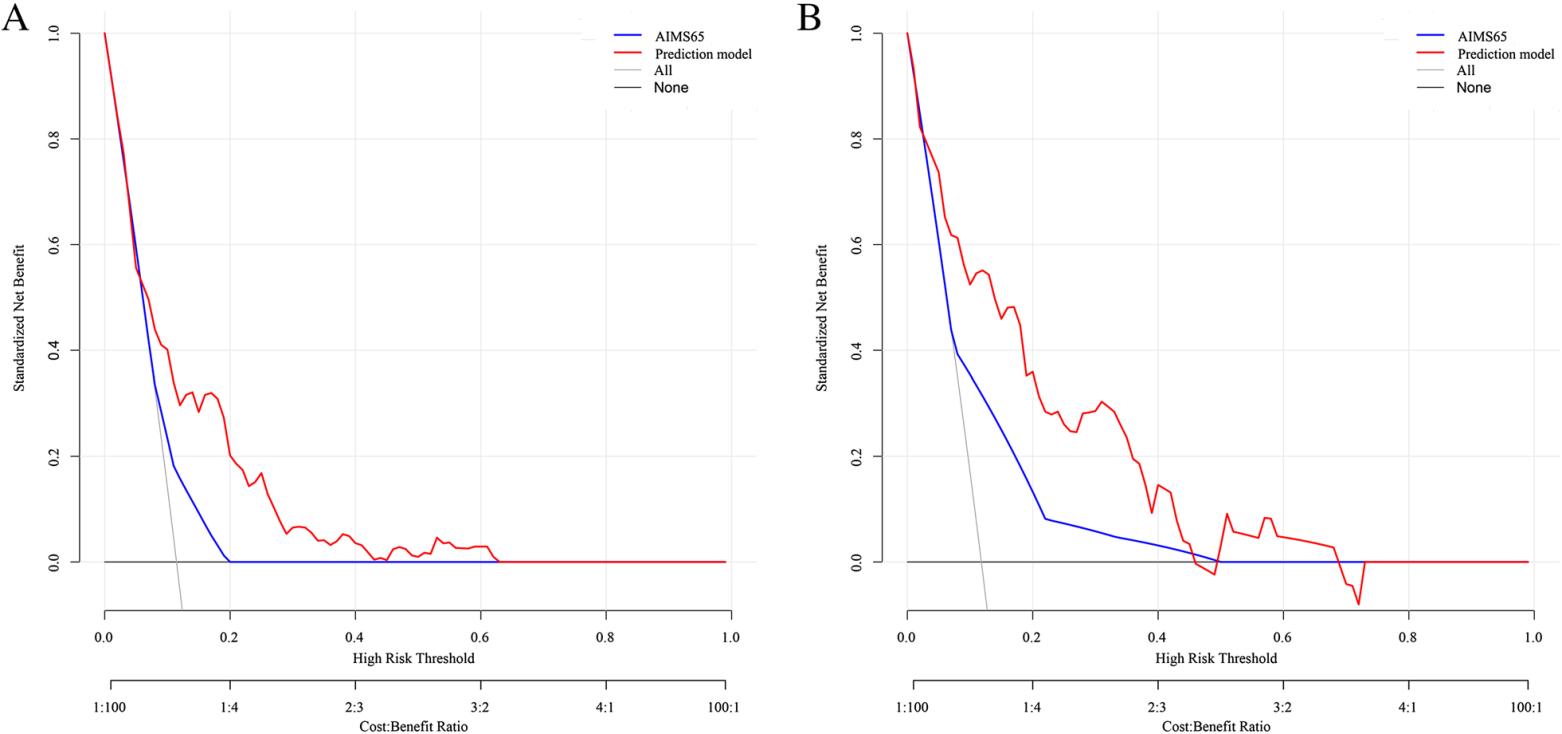
Decision curve analysis (DCA) based on the AIMS65 score system and the prediction model for the training and validation groups. **A** Clinical DCA for the training group. **B** Clinical DCA for the validation group

**Fig. 7:**
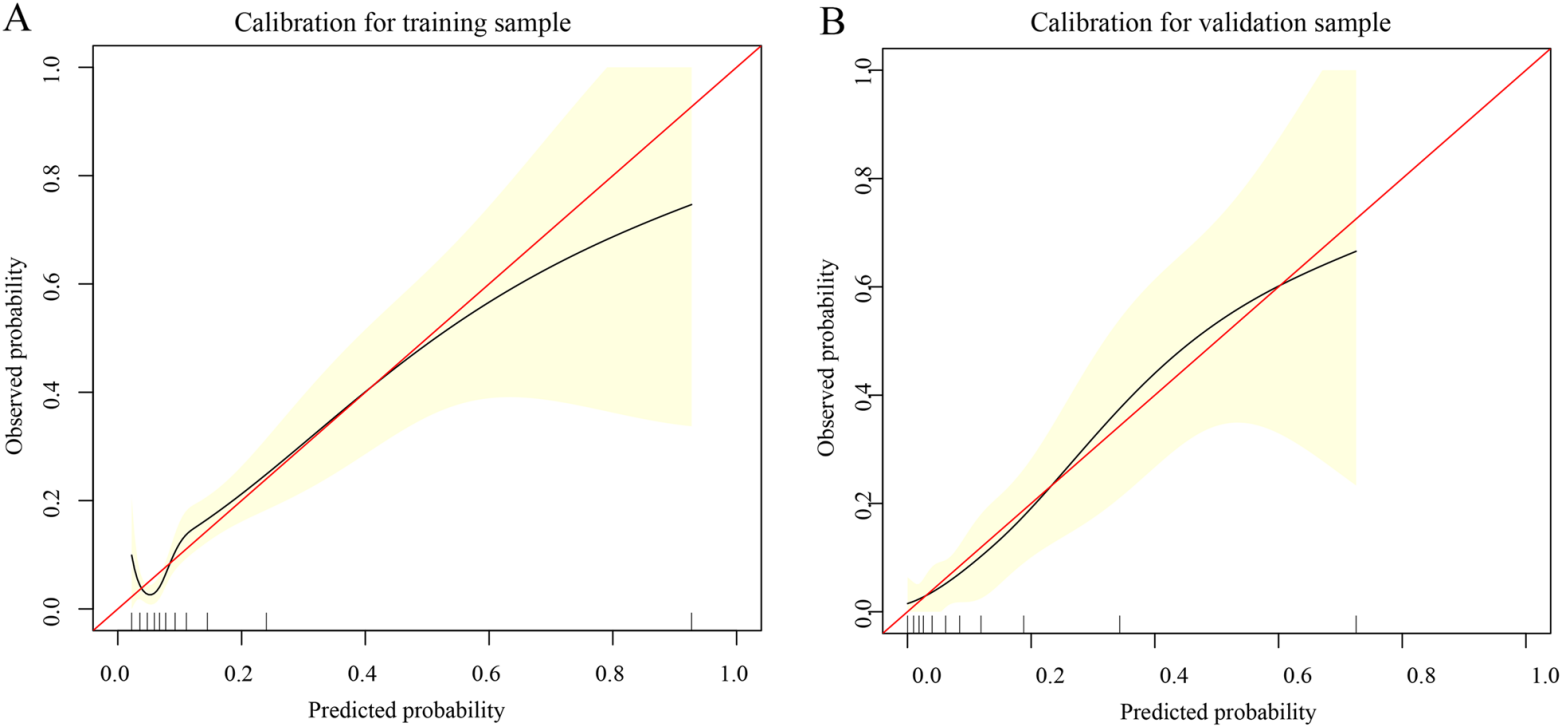
Calibration curve based on the prediction model for the training (**A**) and validation samples (**B**)

To further predict the risk of rebleeding in patients with AUGIB, on the basis of the results of logistic regression analysis for the five risk factors (PLT, D-DIMER, NEUTP, ALB, and LAC), the clinical data of 1170 patients were compiled in R language (version 4.1.0), and a nomogram was drawn by randomly splitting the patient data into the training group (*n* = 899) (Fig. 4A) and the validation group (*n* = 271) (Fig. 4B) in a 3:1 ratio. In the training group, the C index values of our prediction model (including PLT, D-DIMER, NEUTP, ALB, and LAC) and the AIMS65 score model were 0.720 and 0.610, respectively. In the validation group, the C index values of our prediction model and the AIMS65 score model were 0.828 and 0.667, respectively (Fig. 5). The DCA also confirmed that according to nomograms, our predictive model outperformed the AIMS65 scoring system in most cases in both the training and validation groups (Fig. 6). Moreover, the calibration curves of our predictive model nomograms (Fig. 7) showed a good agreement between the predicted and actual outcomes in most cases in both the training and validation groups. In conclusion, our study showed that the new predictive model outperforms the AIMS 65 score model in terms of predictive accuracy, consistency, and net clinical benefit, and our predictive model was validated in both training and validation groups.

## Discussion

In the present study, we analyzed the vital signs, clinical manifestations, and a series of laboratory indicators of patients with AUGIB to determine the risk factors for rebleeding in these patients. Our analysis revealed that LAC, NEUTP, PLT, ALB, and D-DIMER were the significant independent risk factors for rebleeding in AUGIB patients. On the basis of these risk factors, we established a new prediction model and compared its performance with that of the existing AIMS65 score system. The new prediction model could better predict rebleeding occurrence, and thus, this new model could serve as a useful reference in clinical practice.

Some researchers have indicated [13] that the presence of co-morbidities, use of multiple drugs, albumin, and initial presentations with hematemesis can be indicators of rebleeding in patients with UGIB. ALB is the most important protein in human plasma; it maintains body nutrition and osmotic pressure, and it is a sensitive indicator that reflects the overall nutritional status or comprehensive function of the liver. In all patients with UGIB, the required dietary control may lead to malnutrition, as reflected by a low ALB level. In patients with liver cirrhosis, in addition to being used as a monitoring index of liver function, ALB level is also negatively correlated with the degree of esophageal varices; this aspect can be used as a good predictor of esophageal varices bleeding [14]. In the present study, we found that the ALB level in patients with rebleeding was significantly lower than that in patients without rebleeding; this finding suggested that ALB was inversely correlated with the risk of rebleeding and could be used as a valuable predictor of AUGIB.

The NEUTP was significantly higher in the rebleeding group than in the non-rebleeding group. An elevated NEUTP is often observed in patients with infectious diseases, megaloblastic anemia, pernicious anemia, and malignant tumors. Patients with hematological diseases are not included in this group. No significant difference in the white blood cell count was observed between the two groups; thus, the presence of infection might not result in a significant difference. Among the 135 patients in the rebleeding group, 12 patients had gastric cancer, four patients had duodenal cancer, and three patients had esophageal cancer. Patients with malignant tumors accounted for 12.5% of the total patients in the rebleeding group; this proportion was higher than that in the non-rebleeding group (8%). The difference in NEUTP between the two groups may be related to the higher proportion of tumor patients in the rebleeding group.

In a large meta-analysis [15], hemodynamic instability, active endoscopic bleeding, large ulcer size, ulcer location, hemoglobin level, and requirement for blood transfusion were considered to be the main predictors of rebleeding after the first endoscopic hemostatic therapy. In the present study, the LAC value in the rebleeding group was significantly higher than that in the non-rebleeding group. Univariate and multivariate logistic regression analyses also confirmed that LAC was an independent risk factor for rebleeding. LAC is a good indicator of tissue perfusion, and lactate production is increased in patients with severe hypoperfusion and hypoxia. In a retrospective analysis of 114 patients with NVUGIB, Lee et al. [16] found that the lactate clearance rate (LCR) was associated with 30-day rebleeding (odds ratio [OR] 0.931; *P* = 0.033), and LAC as a predictor of clinical outcomes has been confirmed in patients with UGIB [17–19].

The primary role of PLTs is coagulation and hemostasis. The PLT membrane is attached to a plasma layer (the outer covering of PLTs) composed of plasma proteins, coagulation factors, and molecules related to the fibrinolytic system; this membrane plays a role in the hemostasis process after vascular injury. A notable observation is that if the patient’s thrombocytopenia results in prolonged bleeding time, severe injury or rebleeding can occur under stress. In the present study, the PLT level in patients with rebleeding was significantly lower than that in patients without rebleeding. Univariate and multivariate logistic regression analyses also showed that PLT count was a protective factor for rebleeding in patients with AUGIB. The higher the PLT count, the lesser is the possibility of rebleeding occurrence. Bonnet [20] reported that low FIB levels and PLT aggregation dysfunction could predict bleeding risk in patients with decompensated cirrhosis.

Fibrin thrombus formation is important for hemostasis. Increased fibrinolytic activity results in unstable thrombus, which occurs in persistent UGIB and is associated with adverse outcomes [21, 22]. D-DIMER, the main factor that reflects fibrinolytic activity, is a suitable prognostic marker for UGIB [23]. The present study found that D-DIMER level was an independent risk factor for rebleeding after hemostatic treatment and was positively correlated with rebleeding. The higher the D-DIMER level, the higher the possibility of rebleeding in AUGIB patients. Yue et al. [24] also considered that D-DIMER is an independent predictor of rebleeding in patients with UGIB, and its predictive value was superior to the scoring system.

In the univariate analysis, the AIMS65 score of patients in the rebleeding group was significantly higher (*P* < 0.05) than that of the patients in the non-rebleeding group. We constructed a new prediction model comprising five meaningful indicators from the multivariate logistic regression analysis (LAC, NEUTP, PLT, ALB, and D-DIMER). ROC curves were drawn to compare the performance of the AIMS65 score system and the prediction model. The results revealed that the AUC value of the prediction model was greater than that of the AIMS65 score system; this finding indicated that the new prediction model was better than the AIMS65 score system in predicting the risk of rebleeding in patients with AUGIB.

We then predicted the risk of rebleeding in AUGIB patients using the constructed prediction model and randomly divided the data of 1170 patients into the training and validation groups at the ratio of 3:1 to establish a nomogram. In the training group, the C index values of our prediction model and the AIMS65-based model were 0.720 and 0.610, respectively. In the validation group, the C index values of our prediction model and the AIMS65-based model were 0.828 and 0.667, respectively. The decision curve and calibration curve analyses also confirmed that the nomogram based on our prediction model outperformed the AIMS65 scoring system in both training and validation groups, with good consistency in most cases.

To summarize, the prediction model constructed using the five indicators (LAC, NEUTP, PLT, ALB, and D-DIMER) in the present study showed a better predictive value than the AIMS65 score in predicting the risk of rebleeding in patients with AUGIB, and the results were verified in both training and validation groups. Our nomogram showed good predictive performance of the prediction model. We also ranked the predictors according to their importance by machine learning and found that PLT, D-DIMER, and NEUTP were ranked as the top three predictors in the descending order of importance. Clinicians can predict the probability of rebleeding after endoscopic hemostatic therapy in AUGIB patients using this nomogram. This tool should help clinicians to conduct early assessment of patients, early detection of patients who are likely to suffer from rebleeding after endoscopic hemostasis treatment, so as to intervene early, pay more attention to changes in their vital signs, strengthen the application of acid drugs, blood transfusion and volume resuscitation, and perform emergency endoscopy for patients who are likely to suffer from rebleeding. Early interventional examination or surgical intervention should be performed for patients who are difficult to stop bleeding under endoscopy.

Despite demonstrating the predictive value of the predictive model in AUGIB patients, the present study had some limitations. First, this was a retrospective analysis; therefore, some potential factors may have influenced the study findings. Second, although our data were derived from a multicenter study, the number of patients was small, and the present study included indicators of the first test, without dynamic monitoring. Dynamic monitoring of changes of each indicator may provide a more reliable result. Finally, our conclusions require further confirmation through prospective validation studies.

## Conclusion

The present study constructed and validated a new predictive model to predict the probability of rebleeding after endoscopic hemostasis in patients with AUGIB. Combined with laboratory indicators, the model can be conveniently used to identify high-risk patients with rebleeding after endoscopic hemostasis; this can help clinicians to focus more attention on these patients and perform early intervention.

## Data Availability

The data will be made available upon reasonable request to the corresponding authors.
